# X2P-Net: Context-Aware 2D/3D Vertebra Localization

**DOI:** 10.3390/bioengineering13020178

**Published:** 2026-02-03

**Authors:** Rong Tao, Kangqing Ye, Weijun Zhang, Wenyuan Sun, Derong Yu, Donghua Hang, Guoyan Zheng

**Affiliations:** 1 Institute of Medical Robotics, School of Biomedical Engineering, Shanghai Jiao Tong University, Shanghai 200240, China; rongt@nvidia.com (R.T.); yekangq@sjtu.edu.cn (K.Y.); wenyuansun1998@sjtu.edu.cn (W.S.); 1820037839@sjtu.edu.cn (D.Y.); 2 School of Medical Technology, Beijing Institute of Technology, Beijing 100081, China; zhangweijun@tinavi.com; 3 Department of Orthopedics, Shanghai General Hospital, Shanghai Jiao Tong University School of Medicine, Shanghai 200080, China

**Keywords:** spine, vertebra detection, Transformer, attention

## Abstract

In the context of minimally invasive spine surgery, accurately estimating the 3D coordinates of the vertebrae from intraoperative 2D X-ray images is crucial for aligning preoperative data with the patient’s real-time posture. However, existing methods are hindered by the ill-posed nature of 2D-to-3D localization and the distinctive anatomical features of the spinal column, leading to ambiguities and reduced accuracy. In this paper, we introduce X2P-net, a novel prompt-guided and semantic context-enhanced 2D/3D vertebra detection framework. To achieve this, we design a novel Transformer architecture, referred to as BrickFormer, which can automatically extract the refined vertebral foreground context at low computational cost using a dual-attention mechanism. Comprehensive experiments were conducted to validate the proposed approach on two datasets: a large-scale synthetic dataset (BiSpineX) and a sheep spine dataset (SheepSpineX). Results obtained from these experiments demonstrate superior landmark localization performance of the proposed method compared to other state-of-the-art methods. Specifically, on the BiSpineX dataset, X2P-Net achieves percentages of 96.9% and 98.8% at 10 mm and 20 mm thresholds, respectively, a mean position error of 2.99 mm, and an AUC of 0.9923. Similar superior performance was also observed when the proposed method was applied to the SheepSpineX dataset, with percentages of 98.4% and 100.0% at 10 mm and 20 mm thresholds, respectively, a mean position error of 1.08 mm, and an AUC of 0.9972.

## 1. Introduction

Recent years have witnessed an increasing demand for minimally invasive spine surgery (MISS) [1,2]. The success of the surgical procedure depends significantly on the precision with which preoperative planning is mapped onto the patient’s intraoperative position [3]. X-ray fluoroscopy is one of the most popular intraoperative imaging modalities because of its high flexibility, low radiation exposure, and cost-effectiveness. Using intraoperative 2D X-ray images to estimate the 3D locations of the vertebrae—also known as 2D/3D vertebra localization—is crucial for aligning preoperative data with the patient’s intraoperative posture. However, because an X-ray image offers a 2D projected view of the 3D anatomical structures, the 2D/3D vertebra localization task is inherently ill-posed, leading to ambiguities and reduced localization precision [4,5].

Many existing studies have utilized biplanar 2D X-ray images to infer 3D morphological characteristics of the spine [2,6,7,8,9,10,11,12], where the spatial information can be supplemented by two calibrated images acquired from two typical views, i.e., the anteroposterior (AP) and lateral (LAT) views. Currently, two main challenges limit the accuracy of 2D/3D vertebra localization. The first challenge is depth ambiguity, which arises from the superimposition of anatomical structures and the low contrast between the vertebrae and the surrounding soft tissues. The second challenge is semantic ambiguity, which stems from the spine’s chain-like structure, characterized by repetitive vertebrae, making it difficult to distinguish between adjacent vertebrae. Furthermore, metal implants, spinal deformities, and variability in the field of view (FOV) increase the complexity of the task.

**Figure 1:**
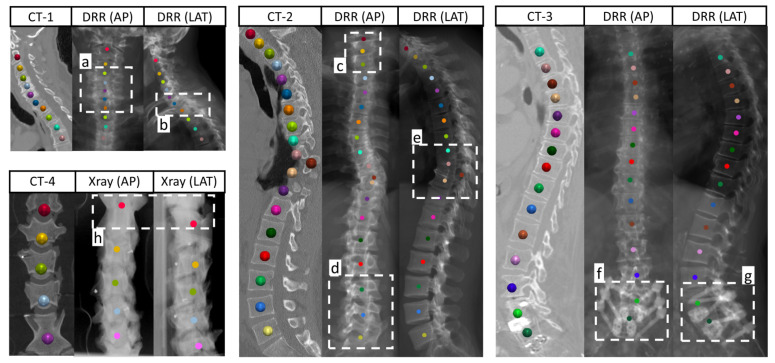
A schematic illustration of the challenges in 2D/3D vertebra localization, where we show four examples, each including the CT volume along with the LAT and AP views of the radiographs. Ground truth 3D vertebral locations are initially annotated on the CT volume and then projected onto 2D image planes. For the first, second, and third examples, we utilize digitally reconstructed radiographs (DRRs) derived from the VerSe dataset [20]; for the fourth example, we show real biplanar X-ray images of the spine of a sheep, captured by a C-arm imaging system. The boxes highlight the common challenges in 2D/3D vertebra localization, including inconsistencies between 2D and 3D annotations (**a**,**c**,**d**), superimposition (**b**), spinal deformities (**e**), metal implants (**f**,**g**), and view-angle disparities (**h**).

To address these challenges, recent studies have proposed various methods that can be largely divided into two categories [13]: lifting-based approaches and direct regression-based approaches. Lifting-based approaches [6,14,15,16,17] involve estimating 2D landmark locations from each view before lifting them into 3D space through triangulation and optimization techniques, such as the least-squares method [16] and statistical shape models [14,15]. Despite its utility, these methods have several limitations, as shown in Figure 1. Notably, 2D landmarks may lack essential depth information for accurate 3D prediction. In particular, when the vertebral centroid landmarks are occluded due to superimposition (Figure 1b), spinal deformities (Figure 1e), or metal implants (Figure 1f,g), it is difficult to estimate precise 3D locations using incomplete visual information. Furthermore, the relationship between 2D visual appearance and 3D structures varies across images. For example, the projection of the vertebral centroid from CT volumes to the 2D detector plane is not always positioned at the center of the projected vertebrae, leading to 2D/3D annotation inconsistency, as demonstrated in Figure 1 a,c,d. Lastly, view-angle disparities between multi-view X-ray images make it difficult to establish one-to-one correspondence between landmarks identified independently on each view, as demonstrated in Figure 1h. On the other hand, direct regression-based methods [6,12,18,19] use 2D visual features from multi-view images to construct a pseudo-3D feature volume, which is then processed by 3D convolutions to regress the voxel-wise likelihood of each landmark. However, the accuracy of these methods is constrained by the spatial resolution of the projected volume, since increasing this resolution leads to a quadratic increase in computational cost.

In this study, we tackle the aforementioned challenges by leveraging advanced semantic context integration strategies to reduce ambiguities and to enhance localization accuracy. First, in contrast to previous works [21,22], which depend only on visual context, we introduce an interactive learning scenario where users can place a point-like prompt on each view to target a reference vertebra. The position and visual features of the reference vertebra are then utilized to enhance the semantic context of the remaining vertebrae. Second, we incorporate the anatomical prior information of the spine through a set of learnable vertebral embeddings. These embeddings are employed to delineate each vertebra using the enhanced 2D features. Subsequently, the delineated 2D vertebral location context is fused with high-resolution features, creating a context-enriched pseudo-3D volume for accurate 3D vertebra localization. To demonstrate the effectiveness of our approach, we designed a context-aware 2D/3D vertebra localization framework that uses biplanar **X**-ray images **to** estimate vertebral **p**ositions in 3D space, hereafter referred to as X2P-Net. The core of X2P-Net is a novel Transformer architecture, referred to as BrickFormer, which facilitates computational efficiency while maintaining performance. Unlike the vanilla Transformer [23], which computes attention weights over all pixels in the feature maps, BrickFormer benefits from a dual-attention mechanism, which automatically discriminates foreground pixels from the background. By removing background pixels, BrickFormer uses sparse foreground pixels as building bricks to support the localization task. Key contributions of this paper are summarized as follows:

- We introduce an end-to-end context-aware 2D/3D vertebra localization framework, referred to as X2P-Net. The framework takes advantage of vertebral context, which is first enhanced by a prompt-guided reference vertebra and then extracted using learnable vertebral embeddings, for high-performing 2D/3D vertebra localization.
- We design a novel BrickFormer architecture, which leverages a dual-attention mechanism. The initial attention layer automatically identifies the foreground region from the background, and the subsequent attention layer then focuses only on the foreground features. This approach achieves high localization accuracy at a low computational cost.
- We conduct comprehensive experiments on two datasets to demonstrate the efficacy of the proposed method: a large-scale synthetic dataset of biplanar digitally reconstructed radiographs (DRRs) and a real biplanar X-ray image dataset of sheep spines, captured by a C-arm imaging system.

## 2. Related Work

### 2.1. Leveraging Semantic Context in Vertebra Localization

Automatic localization of vertebrae from spinal images is a challenging task due to the unique morphology of the spinal column [24]. Published vertebra localization methods have leveraged vertebral context to improve localization accuracy. These methods can be broadly categorized into three groups: statistics-based, context-based, and object detection-based.

In statistics-based approaches, traditional methods employed statistical shape models or atlas-based methods to learn the statistical distribution of all vertebrae [25,26]. Subsequent studies combined machine learning [27] or fully convolutional neural networks [28] with a hidden Markov model (HMM) to identify the locations of vertebral bodies. With recent advancements, deep generative models such as generative adversarial networks (GANs) [29] and normalizing flows [30,31] were proposed to learn the prior distribution of vertebral landmarks. These models were able to implicitly capture the prior distribution, at the cost of additional computational complexity.

Conversely, context-based methods often employ sequential and structural modeling techniques to improve the robustness of vertebra localization by integrating global contextual cues or anatomical priors [32]. For instance, Chen et al. [33] proposed a joint learning model that combined the local appearance of one vertebra and the pairwise conditional dependencies of neighboring vertebrae for vertebra localization from CT images. Similarly, Wang et al. [34] designed an anatomically constrained optimization module that employed a soft constraint to regulate the distance between estimated vertebrae and a hard constraint on the consecutive estimated vertebra labels. Payer et al. [21] proposed a spatial configuration network (SCN-Net) to encode the semantic context of the landmarks. Tao et al. [22] proposed Spine-Transformers that formulated vertebra labeling as a one-to-one set mapping problem and introduced a global loss to incorporate the sequential relationships of vertebrae. Acknowledging the constraints of strict sequential assumptions in prior studies [21,22], especially when handling pathological deformations, researchers have also proposed graph neural networks (GNNs) [35] or reinforcement learning [36] to incorporate anatomical prior information. Specifically, Transformers [23] excel at capturing long-range dependencies, while GNNs and reinforcement learning provide greater flexibility in modeling non-linear spatial relationships and anatomical topologies [37]. For example, Bürgin et al. [38] proposed a hybrid network combining convolutional neural networks (CNNs) and GNNs for robust vertebra identification from CT images. Xiang et al. [39] proposed VLD-Net for localizing and detecting the vertebrae from X-ray images using reinforcement learning with an adaptive exploration mechanism and spine anatomy information.

Recently, object detection models like the You Only Look Once (YOLO) series [40,41,42] and Faster R-CNN [43] have gained increasing adoption for vertebra localization tasks owing to their speed and efficiency. For example, Zhang et al. [44] proposed a method that combined 3D Swin Transformers [45] with YOLOX [41] for accurate spine segment detection from 3D CT images. Huang et al. [46] proposed a method that integrated a bidirectional long short-term memory (LSTM) layer into the Faster R-CNN architecture to implicitly ensure sequential consistency. Although object detector-based approaches showed significant potential in accelerating vertebra localization while maintaining acceptable accuracy, they encountered challenges under severe pathological deformations, primarily due to the lack of explicit anatomical modeling.

A common limitation of the above-mentioned studies on vertebra localization was that they mainly focused on single-image (either a 3D volume or a 2D image) scenarios. However, few studies have addressed the more complex problem of multi-view vertebra localization, where the model not only needs to distinguish each vertebra within each image but also needs to establish cross-view semantic correspondence for landmarks detected on each view. Limited attempts have been made to address this task. For example, Wu et al. [16] proposed a multi-view contrastive learning strategy to capture the anatomical structural information from different views. Huang et al. [9] introduced a multi-perspective network for cross-view vertebra localization, where they used a recurrent module to incorporate contextual information and to enforce anatomical order for the detected vertebrae. While achieving promising results, these methods required either extra post-processing steps or repetitive sampling strategies to assemble the landmarks detected on each view.

### 2.2. Estimating 3D Landmarks from 2D Images

Estimating 3D landmarks from 2D images is inherently an ill-posed problem because multiple 3D predictions may result in the same 2D projection. To alleviate such ambiguities, existing approaches resort to either multi-view visual correspondence [5,47] or long-term temporal clues [48]. As this study focuses on enhancing 3D landmark localization from 2D images for intraoperative applications, we concentrate on studies in the multi-view scenario.

Multi-view images significantly reduce ambiguity in 3D landmark localization, yet effectively aggregating and fusing information from multiple perspectives remains challenging. In the literature, both lifting-based approaches and direct regression-based approaches have been proposed. Lifting-based approaches, such as those by Dong et al. [49] and Bridgeman et al. [50], associate 2D landmark estimations and fuse them into 3D poses. However, establishing cross-view correspondence is still an issue. Existing studies have utilized methods like triangulation [47,51], 4D graph cuts [49], plane sweep stereo [52], cross-view graph matching [53], or statistical shape models [54] to generate 3D poses from 2D landmarks. These methods often adopt a multi-stage framework [55] to refine 3D pose estimation, leading to increased computational cost and accumulated uncertainty. In contrast, direct regression-based approaches [12,47,56,57,58] first build a pseudo-3D feature volume through heatmap estimation and then regress 3D coordinates from the feature volume with 3D CNNs. However, the accuracy of these methods is constrained by the spatial resolution of the projected volume, and increasing this resolution leads to a quadratic increase in computational cost.

## 3. Methodology

### 3.1. Overview

**Figure 2:**
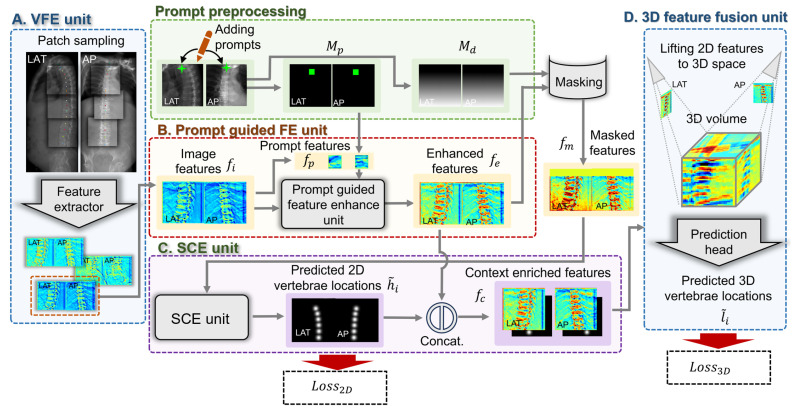
A schematic overview of the proposed framework, consisting of (**A**) a 2D visual feature extraction (VFE) unit; (**B**) a prompt-guided feature enhancement (FE) unit; (**C**) a semantic context extraction (SCE) unit; and (**D**) a 3D multi-view feature fusion unit.

Figure 2 illustrates the network architecture of the proposed context-aware X2P-Net, which consists of a 2D visual feature extraction (VFE) unit, a prompt-guided feature enhancement (FE) unit, a semantic context extraction (SCE) unit, and a 3D multi-view feature fusion unit. The network first utilizes positional information and visual features of the reference vertebra, as indicated by prompts, to enhance the features of the remaining vertebrae. Subsequently, the 2D vertebral context is captured through a series of learnable vertebral embeddings, yielding a set of 2D vertebral heatmaps. These 2D heatmaps are then integrated with high-resolution multi-view image features to construct a pseudo-3D volume, which is used to estimate the 3D coordinates of the vertebrae. Unlike previous 2D/3D landmark localization methods that require pretraining a 2D feature extraction backbone, the proposed method leverages the simultaneous learning of 2D vertebral context and 3D vertebral locations, thereby enabling end-to-end training. Details about each unit are presented below.

### 3.2. The VFE Unit

The VFE unit is shared across both the LAT and the AP views of the spine radiographs. Given a pair of input images, we denote $I_{lat}$ and $I_{ap}$ as the LAT and the AP views, respectively. Each view is represented as $I_{i}\in R^{1\times w\times h}$, where i∈{lat,ap}, and w=512 pixels and h=512 represent the width and height in pixels, respectively. To capture multi-scale visual cues, we utilize a 2D U-Net-like architecture that comprises four levels and a spatial pyramid pooling (SPP) module [59] for feature integration. Subsequently, the 2D VFE unit generates a pair of feature maps corresponding to the respective LAT and AP views, denoted as $\left\{f_{lat},f_{ap}\right\}$, as follows:

(1) $$f_{i}=VFE\left(I_{i}\right),\;\;\forall \;i\in \left\{lat,ap\right\},$$

where $f_{i}\in R^{d\times w\times h}$ and d=64 is the number of feature channels.

In the case where the height of the input images exceeds 512 pixels, we employ a sliding window technique to sample consecutive image patches, as depicted in Figure 2A.

### 3.3. The Prompt-Guided FE Unit

For a given pair of input images, the prompt-guided FE unit requires users to specify a point-like prompt on each image as the input. It is worth noting that the two prompts in the AP and the LAT images need to be placed around the ground truth centers of the same vertebral body, usually the top-most vertebra in both images. These prompts serve as reference points, guiding the network to predict the locations of the vertebral body center for the remaining levels. Similarly, in the case of long-length images, which are divided into a series of contiguous image patches, the bottom-level vertebra from the preceding patch is utilized as the prompt for the subsequent patch. For simplicity, we introduce the prompt-guided FE unit and the SCE unit for a single image patch.

Let us denote the position of the prompt on an image patch as $\left(x_{0},y_{0}\right)$, where $x_{0}$ and $y_{0}$ correspond to the horizontal and vertical coordinates of a point-like prompt, respectively. We first preprocess the input prompt to create two types of masks: a binary prompt mask $M_{p}$ and a unidirectional distance mask $M_{d}$. Specifically, the binary prompt mask $M_{p}\in R^{1\times w\times h}$ is constructed as follows:

(2) $$\begin{array}{c} M_{p}\left(x,y\right)=\left\{\begin{array}{c} 1,\;\left(x,y\right)\in \Omega_{p},\\ 0,\;\mathrm{otherwise}, \end{array}\right.\\ \Omega_{p}=\left\{\left(x,y\right)\;|\;x_{0}-r\leq x<x_{0}+r,\;y_{0}-r\leq y<y_{0}+r\right\}, \end{array}$$

where $\left(x,y\right)$ represents pixel coordinates within the image patch, $\Omega_{p}$ is the set of pixel coordinates covered by the square patch centered at the prompt, and r=40 is an empirical value that delineates a region that can cover the area of the reference vertebral body.

Next, using the features $f_{i}$ from the 2D VFE unit and the prompt mask $M_{p}$, we can obtain prompt features $f_{p}\in R^{d\times 2r\times 2r}$:

(3) $$f_{p}\left(x,y\right)=f_{i}\left(x,y\right)M_{p}\left(x,y\right),\;\;\left(x,y\right)\in \Omega_{p}.$$

Meanwhile, the unidirectional distance mask $M_{d}\in R^{1\times w\times h}$ is derived as follows:

(4) $$M_{d}\left(x,y\right)={∥y-y_{0}∥}_{2},\;\;\forall \left(x,y\right).$$

$M_{d}$ is essential to establish cross-view correspondence, which will be explained in detail in the next section.

**Figure 3:**
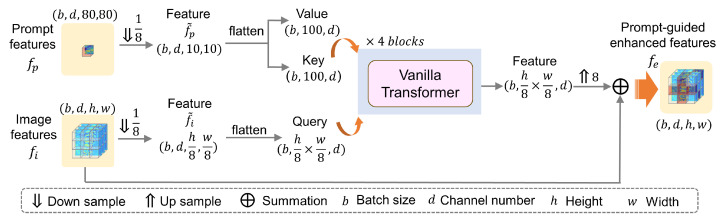
A schematic overview of the prompt-guided FE unit. Using a vanilla Transformer, the image features $f_{i}$ are enhanced with the prompt features $f_{p}$.

Following the preprocessing steps, both $f_{i}$ and $f_{p}$ are down-sampled by a factor of 8, yielding ${\tilde{f}}_{i}$ and ${\tilde{f}}_{p}$, respectively. These down-sampled features are then fed into a 4-layer vanilla Transformer block with 8 heads and a hidden dimension of 64, as depicted in Figure 3. Within the Transformer block, ${\tilde{f}}_{p}$ serves as both the key and the value, which enables the enhancement of repetitive vertebral features present in ${\tilde{f}}_{i}$. The output of the Transformer layer can be presented by the following equation:

(5) $$Out=softmax\left(\frac{\left({\tilde{f}}_{i}W_{Q}\right)\cdot {\left({\tilde{f}}_{p}W_{K}\right)}^{T}}{\sqrt{d}}\right)\cdot \left({\tilde{f}}_{p}W_{V}\right),$$

where $W_{Q}$, $W_{K}$, and $W_{V}$ represent the learnable weight matrices for the query, key, and value, respectively.

Eventually, the prompt-guided FE unit produces a set of enhanced features, represented as $f_{e}\in R^{d\times w\times h}$, which are then fed into the SCE unit to extract the important contextual information of the remaining vertebrae.

### 3.4. The SCE Unit

**Figure 4:**
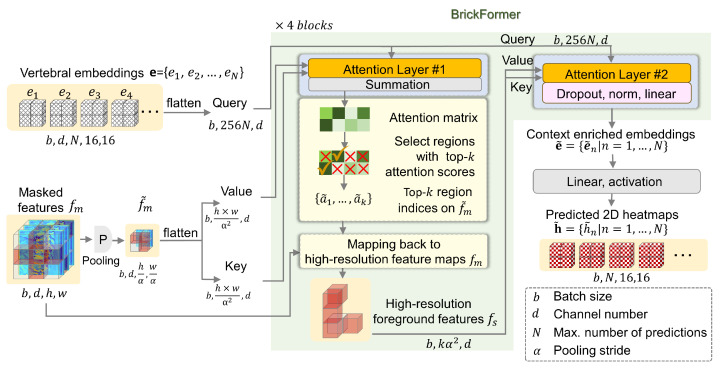
A schematic overview of the SCE unit. Learnable vertebral embeddings capture vertebral context from the masked features through BrickFormer, which automatically delineates the foreground regions.

The SCE unit aggregates spatial contextual information using learnable vertebral embeddings. These embeddings are structured as a sequence of spatial feature maps, denoted as $e=\{e_{n}|n=1,\ldots ,N\}$ with $e_{n}\in R^{d\times 16\times 16}$, where 16×16 is the spatial dimension, and *N* is the maximum number of predicted vertebrae. In conventional Transformers, the computational expense of the attention mechanism increases quadratically with the spatial dimensions of the key and the query matrices. To mitigate this issue, we develop a novel Transformer variant, referred to as BrickFormer, as illustrated in Figure 4. Notably, BrickFormer reduces computational cost by incorporating a dual-attention mechanism. Specifically, in the first attention layer, it automatically delineates the foreground regions from the background using low-resolution feature maps, while in the second attention layer, it refines the process by engaging only the selected high-resolution but sparse foreground features for the extraction of 2D vertebral context.

Mathematically, we first combine the enhanced features $f_{e}$ with the distance mask $M_{d}$ as follows:

(6) $$\begin{array}{c} f_{m}\left(x,y\right)=f_{e}\left(x,y\right){\tilde{M}}_{d}\left(x,y\right)+M_{d}\left(x,y\right),\;\;\forall \left(x,y\right),\\ {\tilde{M}}_{d}\left(x,y\right)=\left\{\begin{array}{c} 0,\;\mathrm{if}\;M_{d}\left(x,y\right)=0,\\ 1,\;\mathrm{if}\;M_{d}\left(x,y\right)>0. \end{array}\right. \end{array}$$

Here, the masked features $f_{m}\in R^{d\times w\times h}$ integrate the vertical distance from each pixel to a reference vertebra indicated by the prompt, thus facilitating spatial alignment of 2D features extracted from different views.

Next, $f_{m}$ undergoes a max pooling operation with a stride of α, obtaining ${\tilde{f}}_{m}$, which is fed into the first attention layer of BrickFormer. Specifically, within the first attention layer, the attention matrix $Attn_{1}$ is calculated as follows:

(7) $$Attn_{1}=softmax\left(\frac{\left(e_{n}W_{Q1}\right)\cdot {\left({\tilde{f}}_{m}W_{K1}\right)}^{T}}{\sqrt{d}}\right),$$

where $W_{Q1}$ and $W_{K1}$ represent the learnable weight matrices for the query and the key, respectively.

Following the attention computation, we sort the elements of the matrix $Attn_{1}$ by their attention scores in descending order. This sorting operation allows us to identify the indices of the top-*k* highest-scoring elements, resulting in a set of indices $\tilde{a}=\left\{{\tilde{a}}_{1},\ldots ,{\tilde{a}}_{k}\right\}$. Then, we map $\tilde{a}$ to the corresponding positions on the high-resolution features $f_{m}$ (one position at the low-resolution features will be mapped to $\alpha^{2}$ positions at the high-resolution features), obtaining the new set of indices $a=\left\{a_{1},\ldots ,a_{k\alpha^{2}}\right\}$, which will be used to extract the subset of foreground features $f_{s}\in R^{d\times k\alpha^{2}}$. Subsequently, these foreground features are input into the second attention layer of BrickFormer for fine-grained attention computation, where the attention matrix $Attn_{2}$ is calculated by the following:

(8) $$Attn_{2}=softmax\left(\frac{\left(e_{n}W_{Q2}\right)\cdot {\left(f_{s}W_{K2}\right)}^{T}}{\sqrt{d}}\right).$$

Finally, the output of BrickFormer is a set of context-enriched vertebral embeddings $\tilde{e}=\left\{{\tilde{e}}_{n}|n=1,\ldots ,N\right\}$ with

(9) $${\tilde{e}}_{n}=Attn_{2}\cdot \left(f_{s}W_{V2}\right),$$

where $W_{Q2}$, $W_{K2}$, and $W_{V2}$ are the corresponding weight matrices for the query, key, and value, respectively.

These embeddings are then fed into linear layers to obtain the predicted 2D vertebral heatmaps $\tilde{h}=\left\{{\tilde{h}}_{n}|n=1,\ldots ,N\right\}$, with ${\tilde{h}}_{n}\in R^{16\times 16}$.

To summarize, in a standard stacked Transformer with *L* attention layers, the complexity of the attention computation is $O\left(LwhMd\right)$, where w×h denotes the spatial dimension of $f_{m}$ and M=16×16×N represents the product of the spatial dimension and the total number of vertebral embeddings. In contrast, our proposed BrickFormer, utilizing the same number of attention layers, significantly reduces the computational complexity to L2OwhMdwhMdα2α2+Okα2Md. Here, the first term corresponds to the attention computation on the low-resolution features, which identifies foreground vertebral regions. The second term represents the fine-grained attention computation on the high-resolution but sparse foreground features, aimed at improving localization accuracy while maintaining low computational cost.

### 3.5. The 3D Multi-View Feature Fusion Unit

The predicted 2D vertebral heatmaps, denoted as ${\tilde{h}}_{i}$, are rescaled to match the input dimensions. These heatmaps are combined with the respective feature maps $f_{e}$ to obtain the concatenated features $f_{c}$, which are then fed into the 3D multi-view feature fusion unit. Within the unit, we unproject the 2D features into a fixed-size pseudo-3D feature volume based on projective geometry. Specifically, we assume that each calibrated view (LAT and AP) is associated with a projection matrix $P_{i}\in R^{3\times 4}$, which is used to project 3D coordinates to 2D image space. During unprojection, each voxel in the pseudo-3D volume is projected onto the 2D image space using the projection matrix. The voxel feature is assigned by sampling the corresponding 2D feature value at the projected location. Then, the volumes from multiple views are aggregated and processed by 3D convolutions to output heatmaps representing 3D vertebral locations. These 3D vertebral heatmaps are subsequently passed through a soft-argmax function, which transforms the heatmaps into precise vertebral coordinates. Finally, the 3D multi-view feature fusion unit produces a set of predicted 3D vertebral coordinates $\left\{{\tilde{l}}_{n}|n=1,\ldots ,N\right\}$ with ${\tilde{l}}_{n}\in R^{1\times 3}$.

### 3.6. Loss Functions

Assuming that the 2D ground truth heatmap for the *n*-th vertebra in view *i* is $h_{i,n}=\left\{p_{i,n}\left(x,y\right)|1⩽x⩽16,\;1⩽y⩽16\right\}$ and the predicted heatmap for each vertebra is ${\tilde{h}}_{i,n}=\left\{{\tilde{p}}_{i,n}\left(x,y\right)|1⩽x⩽16,1⩽y⩽16\right\}$, we compute the MSE loss $Loss_{MSE}$ and Dice loss $Loss_{Dice}$ as follows:

(10) $$Loss_{MSE}=\frac{1}{2\times N\times 16\times 16}\sum_{i}\sum_{n=1}^{N}\sum_{x=1}^{16}\sum_{y=1}^{16}{\left(p_{i,n}\left(x,y\right)-{\tilde{p}}_{i,n}\left(x,y\right)\right)}^{2},$$

and

(11) $$Loss_{Dice}=1-\frac{1}{2N}\sum_{i}\sum_{n=1}^{N}\frac{2\sum_{x=1}^{16}\sum_{y=1}^{16}p_{i,n}\left(x,y\right){\tilde{p}}_{i,n}\left(x,y\right)}{\sum_{x=1}^{16}\sum_{y=1}^{16}{\left(p_{i,n}\left(x,y\right)\right)}^{2}+\sum_{x=1}^{16}\sum_{y=1}^{16}{\left({\tilde{p}}_{i,n}\left(x,y\right)\right)}^{2}},$$

where $p_{i,n}\left(x,y\right)$ and ${\tilde{p}}_{i,n}\left(x,y\right)$ indicate, respectively, the ground truth and the predicted probabilities of a pixel at position $\left(x,y\right)$ for the *n*-th vertebra in view *i*.

Therefore, we obtain the 2D localization loss $Loss_{2D}$ as follows:

(12) $$Loss_{2D}=Loss_{MSE}+Loss_{Dice}.$$

To predict the 3D coordinates of the vertebrae, we assume that the ground truth vertebral locations are $\left\{l_{n}|n=1,\ldots ,N_{v}\right\}$ with $N_{v}\leq N$, where $N_{v}$ is the number of vertebrae present in the current input. We compute an MSE loss $Loss_{3D}$ for each vertebra, as follows:

(13) $$Loss_{3D}=\frac{1}{N_{v}}\sum_{n=1}^{N_{v}}{∥l_{n}-{\tilde{l}}_{n}∥}_{2}^{2}.$$

Then, the overall localization loss $Loss_{Overall}$ is defined as follows:

(14) $$Loss_{Overall}=Loss_{2D}+Loss_{3D}.$$

### 3.7. Implementation Details

The proposed method was developed in Python 3.9 using the PyTorch 2.0 framework and was trained on a workstation equipped with two NVIDIA GeForce RTX 4090 GPUs. The input images had a size of 512×512. The network was trained from scratch in an end-to-end fashion for 100 epochs, employing the AdamW optimizer [60] with a weight decay of 0.05 and a batch size of 2. For the synthetic dataset, we set the maximum number of predicted vertebrae to N=10 and the hyperparameters of BrickFormer to α=32 and k=8. In contrast, for the real sheep spine dataset, we set N=5, α=8, and k=4. These parameter choices were guided by empirical estimates of the foreground-to-background ratio for each dataset.

During the training phase, we incorporate random cropping as a data augmentation technique and take the ground truth centers of the top-most vertebral bodies in both LAT and AP images as prompts. During inference, a point-like prompt is manually placed around the center of the top-most vertebral body in each image. After that, the network simultaneously generates *N* heatmaps for each view and *N* sets of 3D vertebral coordinates. For each predicted heatmap, a vertebra was considered present if the maximum probability $p_{max}$ was higher than 0.5. Thus, we used $p_{max}$ as a criterion to assess the validity of the 3D predictions.

## 4. Experiments

In this section, we present experimental results on a synthetic dataset consisting of biplanar spine DRR images and a real dataset consisting of biplanar sheep spine X-ray images. Below, we first describe the datasets and the evaluation metrics used in our experiments, and then present the experimental results.

### 4.1. Datasets

#### 4.1.1. Synthetic Biplanar Spine DRR Dataset (BiSpineX Dataset)

Given the scarcity of biplanar spine X-ray datasets and the difficulty in obtaining accurate 3D annotations, we generated biplanar DRRs from CT images with precise annotations of vertebral body centroids and created a synthetic dataset, referred to as the BiSpineX dataset. To construct the dataset, we utilized CT volumes from the Large Scale Vertebrae Segmentation Challenge (VerSe) held at MICCAI 2019 and MICCAI 2020 [20]. Since the VerSe dataset included images with a variety of FOVs and resolutions, we excluded cases with mismatched CT volumes and annotations, images with fewer than three vertebrae, and those with file reading errors. This resulted in a dataset of 337 spinal CT volumes, including cases with fractures and metal implants, which were reoriented and resampled to a 1 mm isotropic resolution. For each CT volume, we generated LAT and AP DRRs [61] by simulating X-ray projections using a ray-tracing method that accounts for photon attenuation and scattering. A virtual detector plane of size 0.6×0.6 m^2^ with a resolution of 2048×2048 was used. To address view-angle disparities, we applied random spatial transformations to the CT volumes, including rotations in a range from −15° to 15° about the vertical axis, and from −5° to 5° about both the coronal and sagittal axes. Consequently, we derived 337 pairs of biplanar spine X-ray images from the corresponding CT volumes. The number of vertebrae present in each radiograph ranged from 3 to 24, covering both traditional C-arm X-ray radiographs that typically contain 3 to 5 vertebrae and emerging long-film X-ray images capable of capturing a larger FOV that spans the entire spinal column. The dataset was partitioned into an 80–20% train-test split, with an additional 5% of the training set reserved for validation purposes.

#### 4.1.2. Sheep Spine X-Ray Dataset (SheepSpineX Dataset)

**Figure 5:**
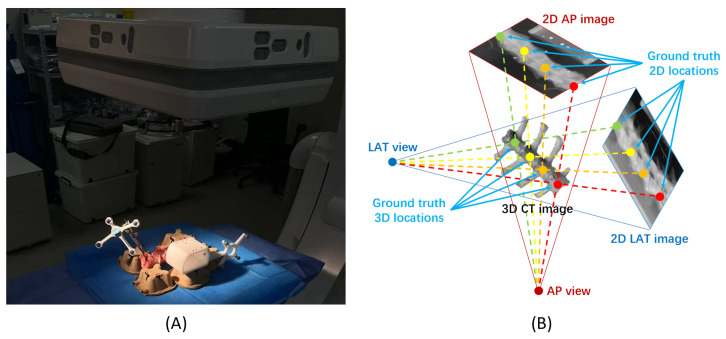
Experimental setup for C-arm image acquisition of a sheep cervical spine. (**A**) Data acquisition of the sheep spine. (**B**) A schematic illustration of the experimental setup where both LAT and AP images were acquired. The 2D landmark ground truth was generated by projecting the 3D landmark ground truth into the 2D image space.

To demonstrate the performance and efficacy of the proposed method, we further conducted experiments on a real sheep spine biplanar X-ray dataset, referred to as the SheepSpineX dataset. This dataset comprises radiographs of 9 sheep cervical spines, which were obtained from a commercial slaughterhouse. For each sheep cervical spine, the radiographs were captured in both AP and LAT views, utilizing a Siemens Arcadis Varic C-Arm system (see Figure 5 for the experimental setup). For each case, we acquired 30 pairs of X-ray images, each pair consisting of LAT and AP views. The number of vertebrae present in each case ranged from 4 to 7. The ground truth 3D vertebral locations were obtained by annotating the centroids of each vertebral body on the corresponding CT volumes. These 3D coordinates were then projected onto the 2D views, yielding precise 2D ground truth vertebral locations. The dataset was divided into 5 cases for training, 1 case for validation, and 3 cases for testing.

### 4.2. Evaluation Metrics

We adopt commonly used metrics [13,20,62] for both 2D and 3D vertebra localization, as follows:

**Percentage of Correct Landmarks (PCL)**: The PCL@τ is defined as the ratio of correctly detected landmarks to the total number of landmarks. A landmark is considered correctly detected if the Euclidean distance to the corresponding ground truth location is below a given threshold τ. PCL@τ is calculated as follows:

(15) $$\begin{array}{c} PCL@\tau =\frac{1}{N_{t}}\sum_{n=1}^{N_{t}}THR_{\tau }\left(l_{n},{\tilde{l}}_{n}\right),\\ THR_{\tau }\left(l_{n},{\tilde{l}}_{n}\right)=\left\{\begin{array}{c} 1,\;\mathrm{if}\;{∥l_{n}-{\tilde{l}}_{n}∥}_{2}<\tau ,\\ 0,\;\mathrm{otherwise}, \end{array}\right. \end{array}$$

where $N_{t}$ denotes the total number of annotated vertebral locations, $l_{n}$ denotes the ground truth location of the *n*-th landmark, and ${\tilde{l}}_{n}$ denotes the corresponding predicted location.

**Mean Position Error (MPE)**: It is defined as the average Euclidean distance, expressed in millimeters (mm) for 3D measurements and pixels for 2D measurements. MPE is calculated as follows:

(16) $$MPE=\frac{1}{N}\sum_{i=0}^{N}{∥x_{i}-{\tilde{x}}_{i}∥}_{2}$$

**Area Under the Curve (AUC)**: It is defined as the area under the PCL curve between the upper limit and the lower limit on the x-axis. This metric is used to evaluate the overall performance of a landmark detection method.

For each of the three metrics, we report the corresponding vertebrae localization performance in both 2D and 3D spaces. Specifically, for the measurement of PCL in 2D, we calculate both $PCL_{2D}@10p$ and $PCL_{2D}@20p$ by setting $\tau_{2D}$ to be 10 pixels and 20 pixels, while for the measurement of PCL in 3D, we compute both $PCL_{3D}@10mm$ and $PCL_{3D}@20mm$ by setting $\tau_{3D}$ to be 10 mm and 20 mm, respectively. Similarly, we estimated $AUC_{2D}$ in 2D by setting the lower limit to be 10 pixels and the upper limit to be 50 pixels, and $AUC_{3D}$ in 3D by setting the lower limit to be 10 mm and the upper limit to be 50 mm.

### 4.3. Results

#### 4.3.1. Results on the BiSpineX Dataset

Due to the limited research on 3D vertebral localization utilizing biplanar radiographs, we evaluated the performance of X2P-Net in comparison with state-of-the-art (SOTA) methods, including benchmark approaches for vertebrae localization, such as SCN-Net [21] and Spine-Transformers (Spine-Trans) [22]. In these methods, 2D vertebral locations are initially identified on the LAT and AP views of radiographs, followed by a triangulation process to derive their 3D coordinates. For the Spine-Transformers, which were initially designed for vertebral localization from CT volumes, we have re-engineered the network and loss functions to adapt them for use with spinal radiographs. Furthermore, we have incorporated classic 2D-3D landmark detection algorithms that have been previously applied to human pose estimation for comparative analysis, including AdaFuse [57], ALG-Net [47], and VOL-Net [47]. All aforementioned methods were trained from the ground up, with methods following the original two-stage training protocol: pretraining the feature extractor, followed by end-to-end fine-tuning of the network.

Results of the comparison study are shown in Table 1. For 3D vertebral landmark localization, X2P-Net achieves the top performance in terms of all evaluation metrics. Specifically, it obtained a $PCL_{3D}@10mm$ of 96.9%, a $PCL_{3D}@20mm$ of 98.8%, an average $MPE_{3D}$ of 2.99 mm, and an $AUC_{3D}$ of 0.9923. In contrast, the second-best method (ALG-Net [47]) obtained a $PCL_{3D}@10mm$ of 95.7%, a $PCL_{3D}@20mm$ of 98.3%, an average $MPE_{3D}$ of 3.25 mm, and an $AUC_{3D}$ of 0.9846. Our method outperforms ALG-Net [47] with a 1.2% increase in $PCL_{3D}@10mm$ and a 0.5% increase in $PCL_{3D}@20mm$, a 0.26 mm decrease in $MPE_{3D}$, and a 0.0077 increase in $AUC_{3D}$.

**Figure 6:**
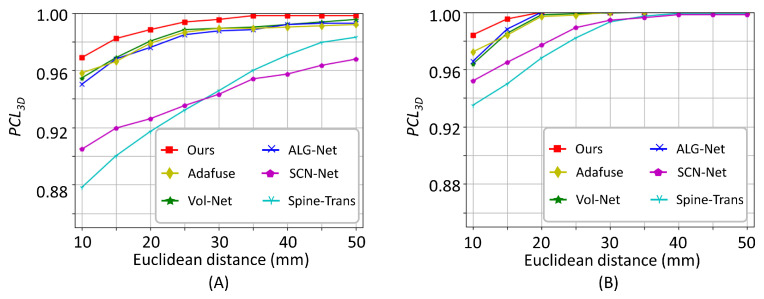
Comparisons of PCL curves from different methods on (**A**) the BiSpineX dataset and (**B**) the SheepSpineX dataset.

Furthermore, we report the 2D vertebra localization performance in Table 1. For X2P-Net, the 2D vertebral locations were derived from the auxiliary output of the predicted vertebral locations for the LAT and AP views. Although our method was not designed for 2D landmark localization, its performance closely matched the best-performing 2D vertebra localization network (SCN-Net [21]), indicating that our method effectively captured the semantic context of each vertebra. Specifically, our method achieved a $PCL_{2D}@20p$ of 96.6% for the LAT view and 96.0% for the AP view, and SCN-Net obtained a $PCL_{2D}@20p$ of 96.9% for the LAT view and 96.8% for the AP view. Thus, compared to SCN-Net, our method exhibited a higher 2D localization error. In particular, our method achieved an average $MPE_{2D}$ of 5.84 pixels for the LAT view and 6.14 pixels for the AP view, while SCN-Net obtained an average $MPE_{2D}$ of 3.78 pixels for the LAT view and 4.96 pixels for the AP view. This higher 2D error is attributed to the lower spatial resolution of our predicted 2D heatmaps. Nonetheless, by integrating the semantic context from 2D landmark predictions with high-resolution 3D features, our method achieved the lowest 3D localization error, demonstrating the efficacy of the proposed method. This efficacy is further illustrated by a visualization of PCL curves in Figure 6A.

**Figure 7:**
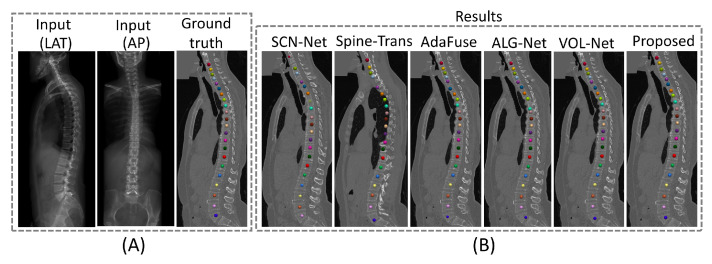
Qualitative comparison of 3D vertebra localization performance across various methods on the BiSpineX dataset. (**A**) One example from the test set of the BiSpineX dataset, showing the CT volume together with the LAT and AP views of the radiographs. Ground truth 3D vertebral locations are annotated on the CT volume. (**B**) 3D vertebra localization results from different methods.

Figure 7 illustrates a challenging case for 2D/3D vertebra localization, where the presence of scoliosis complicates accurate localization. Despite this, our method effectively identifies each vertebra’s location, demonstrating the method’s robustness.

**Figure 8:**
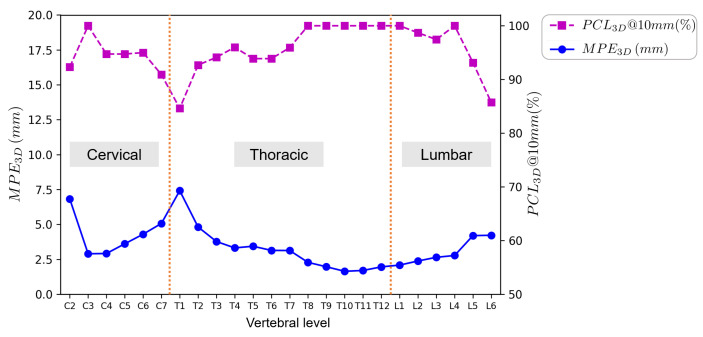
Per-level performance of our method on the BiSpineX dataset measured by $PCL_{3D}@10mm$ and $MPE_{3D}$. The x-axis denotes vertebral levels from the second cervical vertebra to the sixth lumbar vertebra (C2-L6), where letters C, T, and L refer to the cervical, thoracic, and lumbar spine, respectively.

We additionally conducted a per-level analysis of the landmark localization results. We evaluated the per-level landmark localization performance in terms of $PCL_{3D}@10mm$ and $MPE_{3D}$; the results are presented in Figure 8. Localization errors exceeding 10 mm were observed for 35 out of 1123 testing vertebrae. Among these 35 vertebrae, 29 were from cases with scoliosis, and 4 were from cases with metal implants.

#### 4.3.2. Results on the SheepSpineX Dataset

Using the sheep spine dataset, we evaluate X2P-Net against the aforementioned SOTA techniques. The comparative analysis is presented in Table 2, where X2P-Net demonstrates superior performance across all metrics. Specifically, it achieved a $PCL_{3D}@10mm$ of 98.4%, a $PCL_{3D}@20mm$ of 100%, an average $MPE_{3D}$ of 1.08 mm, and an $AUC_{3D}$ of 0.9972. In contrast, the second best-performing method (ALG-Net [47]) attained a $PCL_{3D}@10mm$ of 96.5%, a $PCL_{3D}@20mm$ of 100%, an average $MPE_{3D}$ of 1.56 mm, and an $AUC_{3D}$ of 0.9948. The PCL curves for the various methods are depicted in Figure 6B. Despite influences such as variations in viewing angles, for the sheep spine X-ray images, X2P-Net can still accurately estimate the 3D locations of vertebrae.

### 4.4. Analytical Ablation Studies

We conducted analytical ablation studies on the BiSpineX dataset to evaluate the performance of the proposed X2P-Net. We designed and conducted the following ablation studies: (1) We first performed major component ablations by systematically removing key components to understand their essential role in the landmark localization pipeline. (2) We then conducted a study to compare the proposed BrickFormer with other attention mechanisms. To achieve this, we replaced the BrickFormer attention layers in the network with either vanilla attention layers [23] or sparse attention layers [63] using the same hyperparameter settings (i.e., 4 layers, 8 heads, and a hidden dimension of 512), while keeping the remaining components of the network unchanged. The inputs to these attention layers were the same as those of the first attention layer in BrickFormer. (3) Subsequently, we investigated the impact of different hyperparameters on the performance of our method by systematically varying key hyperparameters while keeping others fixed, to determine the optimal balance between landmark localization accuracy and computational efficiency. (4) Additionally, we investigated the sensitivity of our method to prompt displacement. Specifically, we shifted the prompt along the x- and y-axes in both the LAT and AP images and evaluated performance under these perturbations. (5) Finally, we performed an in-depth analysis of the dual-attention mechanism of BrickFormer by visualizing features at different stages. For each study, the efficiency of each algorithm was quantified by the number of floating-point operations (FLOPs). The larger the FLOPs, the less efficient the algorithm.

#### 4.4.1. Results on Investigating the Effectiveness of Key Components

The results from ablation experiments evaluating the contribution of each unit are shown in Table 3. We explored three network configurations: (1) In the first experiment, named No Prompt, we removed the prompt-guided FE unit, allowing features from the VFE unit to proceed directly to the SCE unit. As shown in Table 3, incorporating the prompt information led to a 6.7% increase in $PCL_{3D}@10mm$, a 5.7% increase in $PCL_{3D}@20mm$, a 3.28 mm decrease in average $MPE_{3D}$, and a 0.0270 increase in $AUC_{3D}$. (2) In the second experiment, named No SCE, we removed both the SCE unit and the $Loss_{2D}$. Consequently, the 3D volumes were solely constructed from the masked features $f_{m}$ from the previous unit. Compared to the No SCE method, our method improved $PCL_{3D}@10mm$ and $PCL_{3D}@20mm$ by 4.6% and 4.0%, respectively, reduced average $MPE_{3D}$ by 2.96 mm, and increased $AUC_{3D}$ by 0.0266. (3) In the last experiment, named No Fusion, we excluded the fusion operation between the predicted 2D vertebral heatmaps and the masked features $f_{m}$. As shown in the results, the No Fusion method achieved better performance than the No SCE method because of the learned vertebral context. However, it still fell short of the performance achieved by our proposed context fusion strategy. Specifically, compared to the No Fusion method, our method achieved 4.1% and 2.3% improvements in $PCL_{3D}@10mm$ and $PCL_{3D}@20mm$, respectively, a 2.91 mm reduction in average $MPE_{3D}$, and a 0.0101 increase in $AUC_{3D}$.

#### 4.4.2. Results on Examining Different Attention Mechanisms

The results of investigating the influence of different attention mechanisms on the performance of the proposed method are presented in Table 4. From this table, one can see that when compared to the vanilla attention mechanism, the proposed BrickFormer demonstrates improved performance with an increase in $PCL_{3D}@10mm$ by 2.2% and $PCL_{3D}@20mm$ by 1.6%, a decrease in average $MPE_{3D}$ by 1.14 mm, and an increase in $AUC_{3D}$ by 0.0129. When compared to the sparse attention mechanism, our method achieved 3.7% and 0.7% improvements in $PCL_{3D}@10mm$ and $PCL_{3D}@20mm$, respectively, a 1.73 mm reduction in average $MPE_{3D}$, and a 0.0053 increase in $AUC_{3D}$. These enhancements are attributed to the dual-attention mechanism embedded within BrickFormer, which effectively filters out irrelevant information from high-resolution foreground features, thereby enhancing localization precision.

#### 4.4.3. Results on Investigating the Impact of Different Hyperparameters

We first examined the impact of the spatial dimensions of the vertebral embeddings on the performance of our proposed method, with dimensions set at 4×4, 8×8, and 16×16, corresponding to the resolution of the predicted 2D vertebral heatmaps. The results are shown in Table 5A. It was evident from the results that the accuracy of vertebra localization improved with an increase in the embedding dimension, peaking at a dimension of 16×16. This enhancement was likely due to the higher resolution of the predicted 2D vertebral heatmaps, which provided more contextual information for improving the precision of 3D vertebra localization. However, increasing the resolution from 4×4 to 16×16 raised the computational cost by approximately 20 million FLOPs, as shown in Table 5A.

Next, we explored the effect of varying the top-*k* value, which was set to 2, 4, or 8, on the performance of our method. The results of this analysis are reported in Table 5B. Increasing the top-*k* from 4 to 8 resulted in a 1.2% and 2.1% increase in $PCL_{3D}@10mm$ and $PCL_{3D}@20mm$, respectively, a reduction in the average $MPE_{3D}$ by 1.2 mm, and an increase in $AUC_{3D}$ by 0.0110. The top-*k* value was directly related to the number of regions selected for fine-grained attention computation during the second stage, with a larger *k* indicating a greater number of features involved in this computation.

In our final hyperparameter ablation study, we investigated the influence of the pooling stride α. As shown in Table 5C, we tested three configurations: α=1, α=2, and α=4. Optimal performance on the BiSpineX dataset was achieved with α=4. Specifically, when α was set to 2 (compared to α=1), $PCL_{3D}$ increased by 1.8% and 1.5% at the 10 mm and 20 mm thresholds, respectively, $MPE_{3D}$ decreased by 0.25 mm, and $AUC_{3D}$ increased by 0.0117. When α was increased to 4, the performance improved, with $PCL_{3D}$ increasing by 2.2% and 1.1% at the respective thresholds, $MPE_{3D}$ decreasing by 1.08 mm, and $AUC_{3D}$ increasing by 0.0117. This improvement was attributed to the fact that a larger α corresponded to a larger receptive field for the attention computation on the low-resolution features and provided more context for fine-grained attention computation, thereby improving localization accuracy.

#### 4.4.4. Results of Investigating the Sensitivity of Our Method to Prompt Displacement

**Figure 9:**
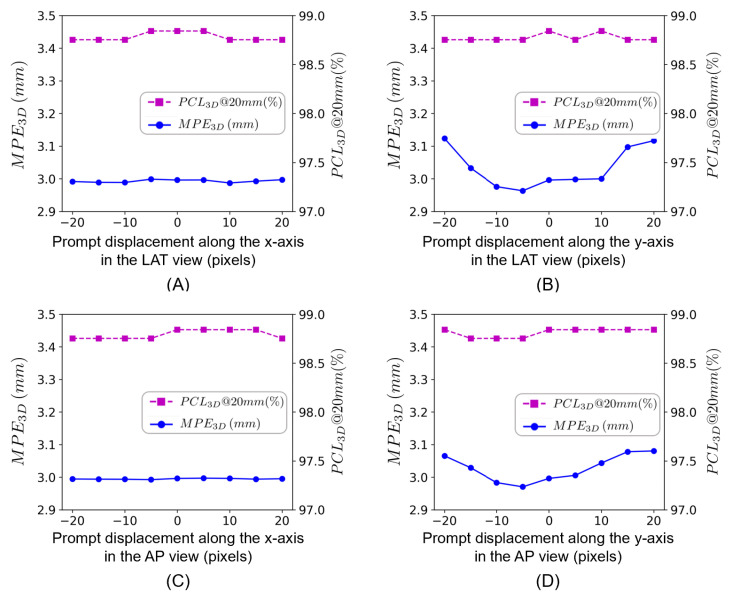
Results of the ablation study evaluating the effect of prompt displacement in terms of $PCL_{3D}@20mm$ and $MPE_{3D}$. The zero point in each sub-figure indicates using the ground truth center as the point-like prompt. (**A**) Results of shifting the prompt along the x-axis in the LAT view. (**B**) Results of shifting the prompt along the y-axis in the LAT view. (**C**) Results of shifting the prompt along the x-axis in the AP view. (**D**) Results of shifting the prompt along the y-axis in the AP view.

To assess the sensitivity of our method to prompt displacement, we investigated the effects of shifting the point-like prompt along the x- and the y-axes (ranging from −20 to +20 pixels away from the ground truth center of the top-most vertebral body in each image) in each image. The performance of the proposed method under different point-like prompt inputs was assessed in terms of $PCL_{3D}@20mm$ and $MPE_{3D}$. Furthermore, to compare the performance when different point-like prompts were used, we conducted one-sided Wilcoxon signed-rank tests [64] and chose a significance level of 0.05. The results of this ablation study are shown in Figure 9. From this figure, one can see that the performance of our method is not sensitive to displacement along the x-axis in both images. In particular, with displacement along the x-axis in either image, the average $MPE_{3D}$ was below 3.00 mm, and the $PCL_{3D}@20mm$ remained above 98.7% for both views. When comparing the results using the ground truth centers with those using the displaced prompts, the maximal change in terms of $MPE_{3D}$ was less than 0.01 mm, and no statistically significant difference was detected (*p*-value = 0.77 for the LAT view and *p*-value = 0.42 for the AP view). However, this is not the case for displacement along the y-axis. In particular, when the displacement was constrained to be 10 pixels around the ground truth center in each image, the maximal change in terms of the average $MPE_{3D}$ increased to 0.06 mm, although no statistically significant difference was detected when comparing the results using the ground truth centers with those using the displaced prompts (*p*-value = 0.09 for the LAT view, *p*-value = 0.83 for the AP view). As the displacement along the y-axis increased further to 20 pixels, the maximal change in terms of the average $MPE_{3D}$ increased to 0.14 mm, and the differences between the results using the ground truth centers and those using the displaced prompts were statistically significant (*p*-values < 0.001 for both views), though the $PCL_{3D}@20mm$ remained above 98.7%.

#### 4.4.5. Analysis of BrickFormer

**Figure 10:**
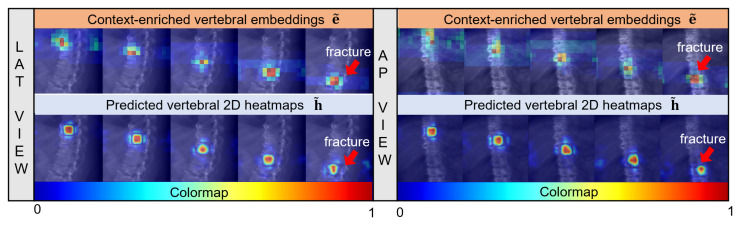
Visualization of features learned from different stages of BrickFormer. Context-enriched vertebral embeddings (the first row) and the predicted 2D vertebral heatmaps (the second row) learned from both the LAT and the AP images of a thoracic spine with fractured vertebrae are displayed. The thoracic spine is taken from the BiSpineX dataset.

To obtain a deeper understanding of the dual-attention mechanism in BrickFormer, we conducted an analysis to determine whether the vertebral embeddings effectively capture vertebral context. To this end, we visualized features at various stages of BrickFormer, as depicted in Figure 10.

Given a pair of input images consisting of LAT and AP views, we visualized the context-enriched vertebral embeddings $\tilde{e}$ (the first row) as well as the predicted 2D vertebral heatmaps $\tilde{h}$ (the second row) of a thoracic spine with a fractured vertebra, which was taken from the BiSpineX dataset. Note that while BrickFormer can handle up to 10 vertebral embeddings, only the first five, corresponding to valid vertebral predictions, were visualized here. Each column represents a single predicted vertebra, ordered from superior to inferior. As illustrated in Figure 10, the locations of the vertebrae can be readily estimated from these context-enriched embeddings, demonstrating the effectiveness of the proposed context-enriched representations.

## 5. Discussions

Estimation of the 3D coordinates of the vertebrae from intraoperative 2D X-ray images is challenging, especially for MISS procedures. This study aimed to develop a novel framework, X2P-Net, that integrated vertebral context to overcome this challenge. Considering the complex and dynamic environment in the operating room, we designed a context-aware 2D/3D vertebra localization framework, which incorporated user interaction in the form of prompts to guide vertebra localization in 3D space. Along with the framework, we introduced BrickFormer, which was a Transformer architecture based on a dual-attention mechanism to delineate each vertebra at low computational cost. The estimated 2D vertebral heatmaps from BrickFormer were fused with multi-view image features to regress 3D vertebral coordinates. Both quantitative and qualitative results demonstrated the effectiveness of the proposed method.

The design of X2P-Net offered several advantages: it leveraged rich vertebral semantics to reduce localization ambiguity and supported a flexible, computationally efficient setup that could be trained end-to-end. This setup ensured high-performance localization in both 2D and 2D/3D scenarios, as demonstrated on synthetic and real spine datasets. Specifically, the prompt-guided FE unit generated masks that extracted the reference vertebral features within specified regions and incorporated positional information for detecting the remaining vertebrae, thereby improving localization performance, as shown in Table 5A. The localization accuracy was further improved by incorporating the proposed dual-attention mechanism in BrickFormer, which could automatically distinguish the foreground regions from the background, as shown in Figure 10.

In comparison with the SOTA methods, X2P-Net achieved better results. Specifically, when evaluated on the BiSpineX dataset, X2P-Net attained a $PCL_{3D}@10mm$ of 96.9% and a $PCL_{3D}@20mm$ of 98.8%, an average $MPE_{3D}$ of 2.99 mm, and an $AUC_{3D}$ of 0.9923. In contrast, the second-best method in terms of $MPE_{3D}$ and $AUC_{3D}$ (ALG-Net [47]) achieved an average $MPE_{3D}$ of 3.25 mm and an $AUC_{3D}$ of 0.9846. Qualitative results shown in Figure 6 and Figure 7 also demonstrate the superior performance of the proposed method. On the SheepSpineX dataset, we observe similarly superior performance of X2P-Net over the SOTA methods [21,22,47,57], with a $PCL_{3D}@10mm$ of 98.4% and a $PCL_{3D}@20mm$ of 100.0%, an $MPE_{3D}$ of 1.08 mm, and an AUC of 0.9972.

Our method was computationally efficient, with 3.24M parameters and 130.8 GMacs. For an input image of size 512 × 512, the average inference time was 0.1 s, with a GPU memory usage of approximately 3 GB. Moreover, in practical clinical workflows, it was typically unnecessary to perform full 3D vertebral localization on every X-ray image [65]; instead, the localization method could be executed on key frames, thereby further reducing computational and memory demands.

It is worth discussing the limitations of the present study. First, although the BiSpineX dataset did involve abnormal cases such as cases with scoliosis or metal implants, and the present method demonstrated superior performance on the BiSpineX dataset, the superiority of the proposed method in more complex clinical scenarios involving more severe abnormal anatomy or more challenging imaging conditions remained to be further validated. Another limitation lies in the requirement of manual placement of a point-like prompt in each view. Results (Figure 9) obtained from the ablation study investigating the sensitivity of the proposed method to the prompt displacement indicated that the proposed method was robust to moderate prompt displacement from the ground truth center (up to 20 pixels along the x-axis and up to 10 pixels along the y-axis) in either view. Thus, in clinical workflows, such prompts are doable with mouse clicks or touchscreen taps [66]. Furthermore, several studies [21,22,37,39,45,46] have shown that one can design an end-to-end network for fully automatic vertebra localization from input X-ray images, which may be used to eliminate the manual placement. Third, potential domain shift arising from differences between synthetic data and real clinical images (e.g., acquisition protocols, anatomy) may affect the generalization of the proposed method to clinical data. Nevertheless, our method was validated on both a synthetic dataset (the BiSpineX dataset) and a real dataset (the SheepSpineX dataset). On both datasets, the proposed method demonstrated better results than the SOTA competing methods, indicating its efficacy in 2D/3D landmark localization.

## 6. Conclusions

In this paper, we introduced X2P-Net, a prompt-guided and context-aware network that estimated the 3D positions of the vertebrae from biplanar X-ray images using a novel BrickFormer architecture. Our network included a prompt-guided FE unit, an SCE unit, and a 3D multi-view feature fusion unit. In addition to visual features, we leveraged vertebral context and positional information from the reference vertebra as indicated by the prompt. We further introduced a generic and novel way to incorporate the anatomical prior information of the spine using a set of learnable vertebral embeddings, which were trained to delineate each vertebral level using BrickFormer. Comprehensive experiments on two datasets demonstrated the superior performance of the proposed method over other SOTA methods. Future work will focus on prospective clinical trials to validate the method in real-world surgical practice. Upon successful validation, X2P-Net could be integrated into MISS systems to support intraoperative guidance, thereby enhancing the safety and quality of spinal surgery.

## Figures and Tables

**Table 1:** Comparisons of 2D and 3D vertebra localization on the BiSpineX dataset with other SOTA methods. ↑: higher value indicates better results. ↓: lower value indicates better results. Pix: pixels. The best results are displayed in bold font.

2D localization (LAT view)				
Methods	$PCL_{2D}@10p\left(\%\right)↑$	$PCL_{2D}@20p\left(\%\right)↑$	$MPE_{2D}\;\left(pix\right)↓$	$AUC_{2D}↑$
SCN-Net [21]	**93.8**	**96.9**	**3.78**	**0.9735**
Spine-Trans [22]	93.2	95.9	3.87	0.9689
AdaFuse [57]	91.1	95.8	4.61	0.9609
ALG-Net [47]	92.5	96.5	4.43	0.9716
VOL-Net [47]	92.3	96.5	4.33	0.9703
Ours	88.2	96.6	5.84	0.9701
2D localization (AP view)				
Methods	$PCL_{2D}@10p\left(\%\right)↑$	$PCL_{2D}@20p\left(\%\right)↑$	$MPE_{2D}\;\left(pix\right)↓$	$AUC_{2D}↑$
SCN-Net [21]	**90.0**	**96.8**	**4.96**	**0.9682**
Spine-Trans [22]	88.1	96.5	5.26	0.9667
AdaFuse [57]	87.6	94.8	5.66	0.9586
ALG-Net [47]	88.6	95.9	5.39	0.9607
VOL-Net [47]	88.2	95.4	5.44	0.9584
Ours	85.2	96.0	6.14	0.9681
3D localization				
Methods	$PCL_{3D}@10mm\left(\%\right)↑$	$PCL_{3D}@20mm\left(\%\right)↑$	$MPE_{3D}\;\left(mm\right)↓$	$AUC_{3D}↑$
SCN-Net [21]	90.5	92.6	8.94	0.9274
Spine-Trans [22]	87.5	91.5	9.21	0.9166
AdaFuse [57]	95.8	97.9	3.95	0.9826
ALG-Net [47]	95.7	98.3	3.25	0.9846
VOL-Net [47]	95.4	98.0	3.49	0.9827
Ours	**96.9**	**98.8**	**2.99**	**0.9923**

**Table 2:** Comparisons of 3D vertebra localization with other SOTA methods on the SheepSpineX dataset. ↑: higher value indicates better results. ↓: lower value indicates better results. The best results are displayed in bold font.

Method	3D Localization			
	${\mathrm{PCL}}_{3D}@10\mathrm{mm}\left(\%\right)$↑	${\mathrm{PCL}}_{3D}@20\mathrm{mm}\left(\%\right)$↑	${\mathrm{MPE}}_{3D}\left(\mathrm{mm}\right)$↓	${\mathrm{AUC}}_{3D}$↑
SCN-Net [21]	95.2	97.2	3.71	0.9854
Spine-Trans [22]	93.5	97.5	4.42	0.9803
AdaFuse [57]	97.2	99.7	2.41	0.9944
ALG-Net [47]	96.5	**100.0**	1.56	0.9948
VOL-Net [47]	96.3	99.8	1.63	0.9939
Ours	**98.4**	**100.0**	**1.08**	**0.9972**

**Table 3:** Results of the ablation study investigating the effectiveness of key components in our method. ↑: higher value indicates better results. ↓: lower value indicates better results. The best results are displayed in bold font.

Components	${\mathrm{PCL}}_{3D}@10\mathrm{mm}\left(\%\right)$↑	${\mathrm{PCL}}_{3D}@20\mathrm{mm}\left(\%\right)$↑	${\mathrm{MPE}}_{3D}\left(\mathrm{mm}\right)$↓	${\mathrm{AUC}}_{3D}$↑	Params ↓	FLOPs ↓
No Prompt	90.2	93.1	6.27	0.9653	3.04 M	129.4 GMac
No SCE	92.3	94.8	5.95	0.9657	**3.00** M	**108.1** GMac
No Fusion	92.8	96.5	5.90	0.9822	3.22 M	123.6 GMac
Ours	**96.9**	**98.8**	**2.99**	**0.9923**	3.24 M	130.8 GMac

**Table 4:** Results of investigating the influence of different attention mechanisms on the performance of the proposed method. ↑: higher value indicates better results. ↓: lower value indicates better results. The best results are displayed in bold font.

Methods	${\mathrm{PCL}}_{3D}@10\mathrm{mm}\left(\%\right)$↑	${\mathrm{PCL}}_{3D}@20\mathrm{mm}\left(\%\right)$↑	${\mathrm{MPE}}_{3D}\left(\mathrm{mm}\right)$↓	${\mathrm{AUC}}_{3D}$↑	Params ↓	FLOPs ↓
Vanilla attention [23]	94.7	97.2	4.13	0.9794	**3.24** M	**109.2** GMac
Sparse attention [63]	93.2	98.1	4.72	0.9870	**3.24** M	111.2 GMac
Ours	**96.9**	**98.8**	**2.99**	**0.9923**	**3.24** M	130.8 GMac

**Table 5:** Results of the ablation study investigating the impact of different hyperparameters. ↑: higher value indicates better results. ↓: lower value indicates better results. The best results are displayed in bold font.

A. Impact of spatial dimensions of vertebral embeddings.						
Dimensions	$PCL_{3D}@10mm\left(\%\right)$↑	$PCL_{3D}@20mm\left(\%\right)$↑	$MPE_{3D}\left(mm\right)$↓	$AUC_{3D}$↑	Params ↓	FLOPs↓
4×4	92.4	95.1	5.92	0.9661	**3.08** M	**109.8** GMac
8×8	93.2	96.7	4.19	0.9731	3.11 M	114.0 GMac
16×16	**96.9**	**98.8**	**2.99**	**0.9923**	3.24 M	130.8 GMac
B. Impact of top-*k* value.						
Top-*k*	$PCL_{3D}@10mm\left(\%\right)$↑	$PCL_{3D}@20mm\left(\%\right)$↑	$MPE_{3D}\left(mm\right)$↓	$AUC_{3D}$↑	Params ↓	FLOPs ↓
2	93.1	95.4	5.63	0.9665	**3.24** M	**114.7** GMac
4	95.7	97.9	3.59	0.9813	**3.24** M	120.0 GMac
8	**96.9**	**98.8**	**2.99**	**0.9923**	**3.24** M	130.8 GMac
C. Impact of pooling stride α.						
α	$PCL_{3D}@10mm\left(\%\right)$↑	$PCL_{3D}@20mm\left(\%\right)$↑	$MPE_{3D}\left(mm\right)$↓	$AUC_{3D}$↑	Params ↓	FLOPs ↓
1	92.9	96.2	4.32	0.9689	**3.24** M	**110.8** GMac
2	94.7	97.7	4.07	0.9806	**3.24** M	114.7 GMac
4	**96.9**	**98.8**	**2.99**	**0.9923**	**3.24** M	130.8 GMac

## Data Availability

The original contributions presented in this study are included in the article. Further inquiries can be directed to the corresponding authors.

## References

[1] Tajsic T., Patel K., Farmer R., Mannion R., Trivedi R. (2018). Spinal navigation for minimally invasive thoracic and lumbosacral spine fixation: Implications for radiation exposure, operative time, and accuracy of pedicle screw placement. Eur. Spine J..

[2] Maken P., Gupta A. (2023). 2D-to-3D: A review for computational 3D image reconstruction from X-ray images. Arch. Comput. Methods Eng..

[3] Unberath M., Gao C., Hu Y., Judish M., Taylor R.H., Armand M., Grupp R. (2021). The impact of machine learning on 2d/3d registration for image-guided interventions: A systematic review and perspective. Front. Robot. AI.

[4] Drover D., MV R., Chen C.H., Agrawal A., Tyagi A., Huynh C.P. (2018). Can 3D Pose Be Learned from 2D Projections Alone?. Proceedings of the European Conference on Computer Vision, Munich, Germany, 8–14 September 2018.

[5] Zhao Q., Zheng C., Liu M., Chen C. (2024). A single 2d pose with context is worth hundreds for 3d human pose estimation. Adv. Neural Inf. Process. Syst..

[6] Aubert B., Vazquez C., Cresson T., Parent S., de Guise J.A. (2019). Toward automated 3D spine reconstruction from biplanar radiographs using CNN for statistical spine model fitting. IEEE Trans. Med. Imaging.

[7] Wang L., Xu Q., Leung S., Chung J., Chen B., Li S. (2019). Accurate automated Cobb angles estimation using multi-view extrapolation net. Med. Image Anal..

[8] Kasten Y., Doktofsky D., Kovler I. (2020). End-to-end convolutional neural network for 3D reconstruction of knee bones from bi-planar X-ray images. Proceedings of the Machine Learning for Medical Image Reconstruction: Third International Workshop, MLMIR 2020, Held in Conjunction with MICCAI 2020, Lima, Peru, 8 October 2020.

[9] Huang Y., Jones C.K., Zhang X., Johnston A., Waktola S., Aygun N., Witham T., Bydon A., Theodore N., Helm P.A. (2022). Multi-perspective region-based CNNs for vertebrae labeling in intraoperative long-length images. Comput. Methods Programs Biomed..

[10] Kyung D., Jo K., Choo J., Lee J., Choi E. (2023). Perspective projection-based 3d CT reconstruction from biplanar X-rays. Proceedings of the ICASSP 2023-2023 IEEE International Conference on Acoustics, Speech and Signal Processing (ICASSP).

[11] Cafaro A., Spinat Q., Leroy A., Maury P., Munoz A., Beldjoudi G., Robert C., Deutsch E., Grégoire V., Lepetit V. (2023). X2Vision: 3D CT Reconstruction from Biplanar X-Rays with Deep Structure Prior. Proceedings of the International Conference on Medical Image Computing and Computer-Assisted Intervention.

[12] Ye K., Sun W., Tao R., Zheng G. (2025). A Projective-Geometry-Aware Network for 3D Vertebra Localization in Calibrated Biplanar X-Ray Images. Sensors.

[13] Wang J., Tan S., Zhen X., Xu S., Zheng F., He Z., Shao L. (2021). Deep 3D human pose estimation: A review. Comput. Vis. Image Underst..

[14] Zheng G., Gollmer S., Schumann S., Dong X., Feilkas T., Ballester M.A.G. (2009). A 2D/3D correspondence building method for reconstruction of a patient-specific 3D bone surface model using point distribution models and calibrated X-ray images. Med. Image Anal..

[15] Baka N., Kaptein B.L., de Bruijne M., van Walsum T., Giphart J., Niessen W.J., Lelieveldt B.P. (2011). 2D–3D shape reconstruction of the distal femur from stereo X-ray imaging using statistical shape models. Med. Image Anal..

[16] Wu H., Zhang J., Fang Y., Liu Z., Wang N., Cui Z., Shen D. (2023). Multi-view vertebra localization and identification from ct images. Proceedings of the International Conference on Medical Image Computing and Computer-Assisted Intervention.

[17] Kim H., Lee K., Lee D., Baek N. (2019). 3D reconstruction of leg bones from X-ray images using CNN-based feature analysis. Proceedings of the 2019 International Conference on Information and Communication Technology Convergence (ICTC).

[18] Aubert B., Vidal P., Parent S., Cresson T., Vazquez C., De Guise J. (2017). Convolutional neural network and in-painting techniques for the automatic assessment of scoliotic spine surgery from biplanar radiographs. Proceedings of the Medical Image Computing and Computer-Assisted Intervention- MICCAI 2017: 20th International Conference, Quebec City, QC, Canada, 11–13 September 2017.

[19] Bayat A., Sekuboyina A., Paetzold J.C., Payer C., Stern D., Urschler M., Kirschke J.S., Menze B.H. (2020). Inferring the 3D standing spine posture from 2D radiographs. Proceedings of the Medical Image Computing and Computer Assisted Intervention–MICCAI 2020: 23rd International Conference, Lima, Peru, 4–8 October 2020.

[20] Sekuboyina A., Husseini M.E., Bayat A., Löffler M., Liebl H., Li H., Tetteh G., Kukačka J., Payer C., Štern D. (2021). VerSe: A vertebrae labelling and segmentation benchmark for multi-detector CT images. Med. Image Anal..

[21] Payer C., Štern D., Bischof H., Urschler M. (2019). Integrating spatial configuration into heatmap regression based CNNs for landmark localization. Med. Image Anal..

[22] Tao R., Liu W., Zheng G. (2022). Spine-transformers: Vertebra labeling and segmentation in arbitrary field-of-view spine CTs via 3D transformers. Med. Image Anal..

[23] Vaswani A., Shazeer N., Parmar N., Uszkoreit J., Jones L., Gomez A.N., Kaiser Ł., Polosukhin I. (2017). Attention is all you need. Adv. Neural Inf. Process. Syst..

[24] Xie Z., Lin Z., Sun E., Ding F., Qi J., Zhao S. (2025). Deep learning for automatic vertebra analysis: A methodological survey of recent advances. Comput. Med. Imaging Graph..

[25] Klinder T., Ostermann J., Ehm M., Franz A., Kneser R., Lorenz C. (2009). Automated model-based vertebra detection, identification, and segmentation in CT images. Med. Image Anal..

[26] Schmidt S., Kappes J., Bergtholdt M., Pekar V., Dries S., Bystrov D., Schnörr C. (2007). Spine detection and labeling using a parts-based graphical model. Proceedings of the Information Processing in Medical Imaging: 20th International Conference, IPMI 2007, Kerkrade, The Netherlands, 2–6 July 2007.

[27] Glocker B., Zikic D., Konukoglu E., Haynor D.R., Criminisi A. (2013). Vertebrae localization in pathological spine CT via dense classification from sparse annotations. Proceedings of the Medical Image Computing and Computer-Assisted Intervention–MICCAI 2013: 16th International Conference, Nagoya, Japan, 22–26 September 2013.

[28] Chen Y., Gao Y., Li K., Zhao L., Zhao J. (2019). Vertebrae identification and localization utilizing fully convolutional networks and a hidden Markov model. IEEE Trans. Med. Imaging.

[29] Han Z., Wei B., Mercado A., Leung S., Li S. (2018). Spine-GAN: Semantic segmentation of multiple spinal structures. Med. Image Anal..

[30] Huang Z., Zhao R., Leung F.H., Banerjee S., Lam K.M., Zheng Y.P., Ling S.H. (2024). Landmark Localization from Medical Images with Generative Distribution Prior. IEEE Trans. Med. Imaging.

[31] Ye K., Zou X., Sun W., Zheng G. (2025). Semi-GDE: Generative distribution estimation for semi-supervised medical landmark localization. Neurocomputing.

[32] Yang Y., Wang Y., Liu T., Wang M., Sun M., Song S., Fan W., Huang G. (2025). Anatomical prior-based vertebral landmark detection for spinal disorder diagnosis. Artif. Intell. Med..

[33] Chen H., Shen C., Qin J., Ni D., Shi L., Cheng J.C., Heng P.A. (2015). Automatic localization and identification of vertebrae in spine CT via a joint learning model with deep neural networks. Proceedings of the Medical Image Computing and Computer-Assisted Intervention–MICCAI 2015: 18th International Conference, Munich, Germany, 5–9 October 2015.

[34] Wang F., Zheng K., Lu L., Xiao J., Wu M., Miao S. (2021). Automatic vertebra localization and identification in CT by spine rectification and anatomically-constrained optimization. Proceedings of the IEEE/CVF Conference on Computer Vision and Pattern Recognition.

[35] Scarselli F., Gori M., Tsoi A.C., Hagenbuchner M., Monfardini G. (2008). The graph neural network model. IEEE Trans. Neural Netw..

[36] Mnih V., Kavukcuoglu K., Silver D., Rusu A.A., Veness J., Bellemare M.G., Graves A., Riedmiller M., Fidjeland A.K., Ostrovski G. (2015). Human-level control through deep reinforcement learning. Nature.

[37] Chen D., Chen M., Wu P., Wu M., Zhang T., Li C. (2025). Two-stream spatio-temporal GCN-transformer networks for skeleton-based action recognition. Sci. Rep..

[38] Bürgin V., Prevost R., Stollenga M.F. (2023). Robust vertebra identification using simultaneous node and edge predicting graph neural networks. Proceedings of the International Conference on Medical Image Computing and Computer-Assisted Intervention.

[39] Xiang S., Zhang L., Wang Y., Zhou S., Zhao X., Zhang T., Li S. (2025). VLD-Net: Localization and Detection of the Vertebrae from X-ray Images by Reinforcement Learning with Adaptive Exploration Mechanism and Spine Anatomy Information. IEEE J. Biomed. Health Inform..

[40] Redmon J., Divvala S., Girshick R., Farhadi A. (2016). You only look once: Unified, real-time object detection. Proceedings of the IEEE Conference on Computer Vision and Pattern Recognition.

[41] Ge Z., Liu S., Wang F., Li Z., Sun J. (2021). Yolox: Exceeding yolo series in 2021. arXiv.

[42] Wang C.Y., Yeh I.H., Mark Liao H.Y. (2024). Yolov9: Learning what you want to learn using programmable gradient information. Proceedings of the European Conference on Computer Vision.

[43] Ren S., He K., Girshick R., Sun J. (2016). Faster R-CNN: Towards real-time object detection with region proposal networks. IEEE Trans. Pattern Anal. Mach. Intell..

[44] Zhang Y., Ji X., Liu W., Li Z., Zhang J., Liu S., Zhong W., Hu L., Li W. (2023). A spine segmentation method under an arbitrary field of view based on 3d swin transformer. Int. J. Intell. Syst..

[45] Liu Z., Lin Y., Cao Y., Hu H., Wei Y., Zhang Z., Lin S., Guo B. (2021). Swin transformer: Hierarchical vision transformer using shifted windows. Proceedings of the IEEE/CVF International Conference on Computer Vision.

[46] Huang Y., Jones C.K., Zhang X., Johnston A., Aygun N., Witham T., Helm P.A., Siewerdsen J.H., Uneri A. (2022). Automatic labeling of vertebrae in long-length intraoperative imaging with a multi-view, region-based CNN. Proceedings of the Medical Imaging 2022: Image-Guided Procedures, Robotic Interventions, and Modeling.

[47] Iskakov K., Burkov E., Lempitsky V., Malkov Y. (2019). Learnable triangulation of human pose. Proceedings of the IEEE/CVF International Conference on Computer Vision.

[48] Zheng C., Zhu S., Mendieta M., Yang T., Chen C., Ding Z. (2021). 3d human pose estimation with spatial and temporal transformers. Proceedings of the IEEE/CVF International Conference on Computer Vision.

[49] Dong J., Jiang W., Huang Q., Bao H., Zhou X. (2019). Fast and robust multi-person 3d pose estimation from multiple views. Proceedings of the IEEE/CVF Conference on Computer Vision and Pattern Recognition.

[50] Bridgeman L., Volino M., Guillemaut J.Y., Hilton A. (2019). Multi-Person 3D Pose Estimation and Tracking in Sports. Proceedings of the 2019 IEEE/CVF Conference on Computer Vision and Pattern Recognition Workshops (CVPRW).

[51] Ju F., Wang Y., Zhao J., Dong M. (2025). Multiview 2D/3D image registration in minimally invasive pelvic surgery navigation. Sci. Rep..

[52] Lin J., Lee G.H. (2021). Multi-view multi-person 3d pose estimation with plane sweep stereo. Proceedings of the IEEE/CVF Conference on Computer Vision and Pattern Recognition.

[53] Wu S., Jin S., Liu W., Bai L., Qian C., Liu D., Ouyang W. (2021). Graph-based 3d multi-person pose estimation using multi-view images. Proceedings of the IEEE/CVF International Conference on Computer Vision.

[54] Bogo F., Kanazawa A., Lassner C., Gehler P., Romero J., Black M.J. (2016). Keep it SMPL: Automatic estimation of 3D human pose and shape from a single image. Proceedings of the Computer Vision–ECCV 2016: 14th European Conference, Amsterdam, The Netherlands, 11–14 October 2016.

[55] Tome D., Toso M., Agapito L., Russell C. (2018). Rethinking pose in 3d: Multi-stage refinement and recovery for markerless motion capture. Proceedings of the 2018 International Conference on 3D Vision (3DV).

[56] Tu H., Wang C., Zeng W. (2020). Voxelpose: Towards multi-camera 3d human pose estimation in wild environment. Proceedings of the Computer Vision–ECCV 2020: 16th European Conference, Glasgow, UK, 23–28 August 2020.

[57] Zhang Z., Wang C., Qiu W., Qin W., Zeng W. (2021). Adafuse: Adaptive multiview fusion for accurate human pose estimation in the wild. Int. J. Comput. Vis..

[58] Ye H., Zhu W., Wang C., Wu R., Wang Y. (2022). Faster voxelpose: Real-time 3d human pose estimation by orthographic projection. Proceedings of the European Conference on Computer Vision.

[59] He K., Zhang X., Ren S., Sun J. (2015). Spatial pyramid pooling in deep convolutional networks for visual recognition. IEEE Trans. Pattern Anal. Mach. Intell..

[60] Zhou P., Xie X., Lin Z., Yan S. (2024). Towards understanding convergence and generalization of AdamW. IEEE Trans. Pattern Anal. Mach. Intell..

[61] Russakoff D.B., Rohlfing T., Mori K., Rueckert D., Ho A., Adler J.R., Maurer C.R. (2005). Fast generation of digitally reconstructed radiographs using attenuation fields with application to 2D-3D image registration. IEEE Trans. Med. Imaging.

[62] Guo X., Xu S., Lin X., Sun Y., Ma X. (2022). 3D hand pose estimation from a single RGB image through semantic decomposition of VAE latent space. Pattern Anal. Appl..

[63] Zaheer M., Guruganesh G., Dubey K.A., Ainslie J., Alberti C., Ontanon S., Pham P., Ravula A., Wang Q., Yang L. (2020). Big bird: Transformers for longer sequences. Adv. Neural Inf. Process. Syst..

[64] Wilcoxon F. (1992). Individual comparisons by ranking methods. Breakthroughs in Statistics: Methodology and Distribution.

[65] Brost A., Liao R., Strobel N., Hornegger J. (2010). Respiratory motion compensation by model-based catheter tracking during EP procedures. Med. Image Anal..

[66] Niu K., Tao Z., Cheng L., Wei Z., Kang H., Wei T., Huang B., Xu F., Xiong C. (2025). Comprehensive workflow with optical navigation in minimally invasive transforaminal lumbar interbody fusion: A retrospective study. J. Orthop. Surg. Res..

